# Spatial neglect

**DOI:** 10.1136/practneurol-2015-001115

**Published:** 2015-05-28

**Authors:** Korina Li, Paresh A Malhotra

**Affiliations:** 1 Centre for Restorative Neuroscience, Division of Brain Sciences, Imperial College London, London, UK; 2 Department of Neurology, University Hospital Coventry, Coventry, West Midlands, UK

**Keywords:** NEGLECT, COGNITION, STROKE, ATTENTION

## Abstract

The syndrome of visuospatial neglect is a common consequence of unilateral brain injury. It is most often associated with stroke and is more severe and persistent following right hemisphere damage, with reported frequencies in the acute stage of up to 80%. Neglect is primarily a disorder of attention whereby patients characteristically fail to orientate, to report or to respond to stimuli located on the contralesional side. Neglect is usually caused by large strokes in the middle cerebral artery territory and is heterogeneous, such that most patients do not manifest every feature of the syndrome. A number of treatments may improve neglect, but there is no widely accepted universal approach to therapy. Although most patients recover spontaneously, the evidence suggests that they continue to have significant cognitive impairments, particularly relating to attention.

## Introduction

The syndrome of spatial neglect is relatively common. Several pathological processes may cause it, including neurodegenerative disease,^1^ ^2^ neoplasia^3^ and trauma,^4^ although it is most common in the context of hemispheric stroke.^5^ Because of its implications for the understanding of the perception and representation of space, neglect has been of considerable interest to neuroscientists, psychologists and philosophers.^6–8^ However, it is also very important to clinicians as it may profoundly affect recovery from stroke; indeed, neglect's negative effects on rehabilitation outcome may be even greater than those of hemiplegia.^9^ ^10^ Neglect may follow right hemisphere stroke in up to 82% of patients^5^ in the acute stage, but most studies describe rates closer to 50%.^11^

The terms unilateral neglect, hemineglect and spatial neglect are used interchangeably. They are generally defined as an inability to perceive, report and orient to sensory events towards one side of space, contralateral to the side of the lesion, with or without a primary sensory deficit.^12^ Neglect is more common and longer-lasting after right hemisphere stroke, most likely because of the right hemisphere's key role in attentional processes; thus, most of the discussion below refers to neglect for the left side of space.^13^

## Neglect and extinction

**Figure 1:**
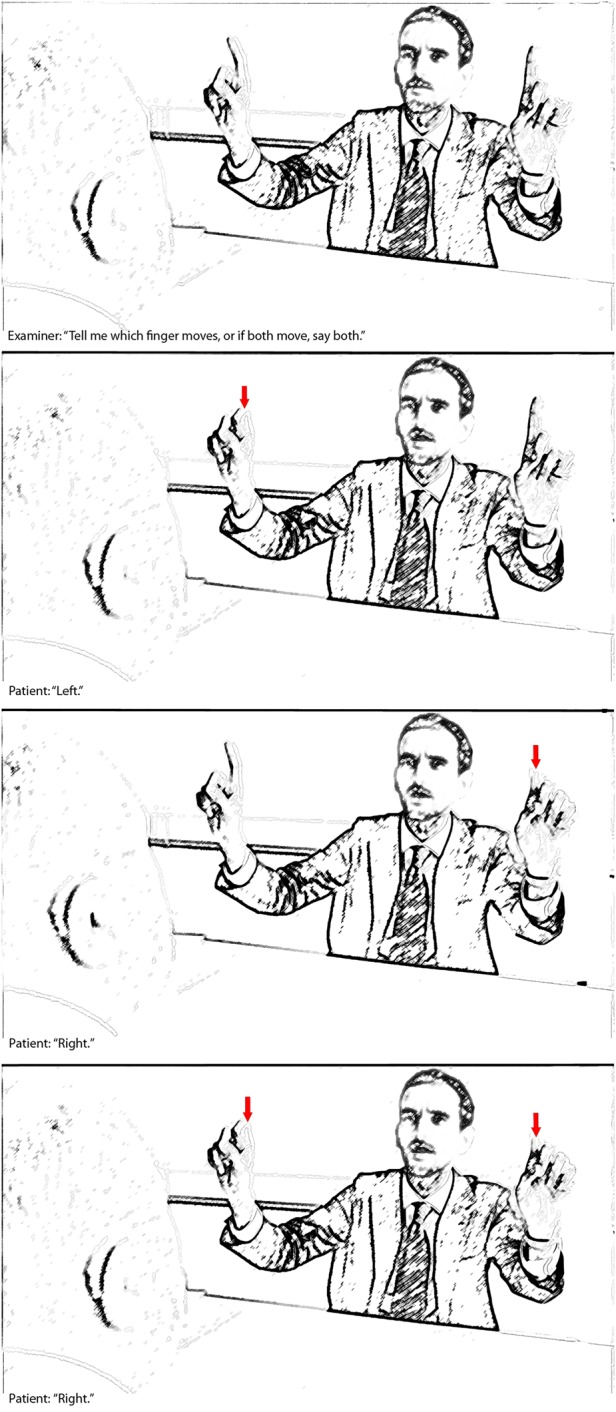
Demonstration of visual extinction. The patient can report the left-sided (middle panel) stimulus and the right-sided stimulus (top panel) when they are presented on their own, but reports only the rightward stimulus when both are presented together (bottom panel). Note that in the video examination the patient manifests left-sided extinction as well as a left-sided hemianopia.

**Figure 2:**
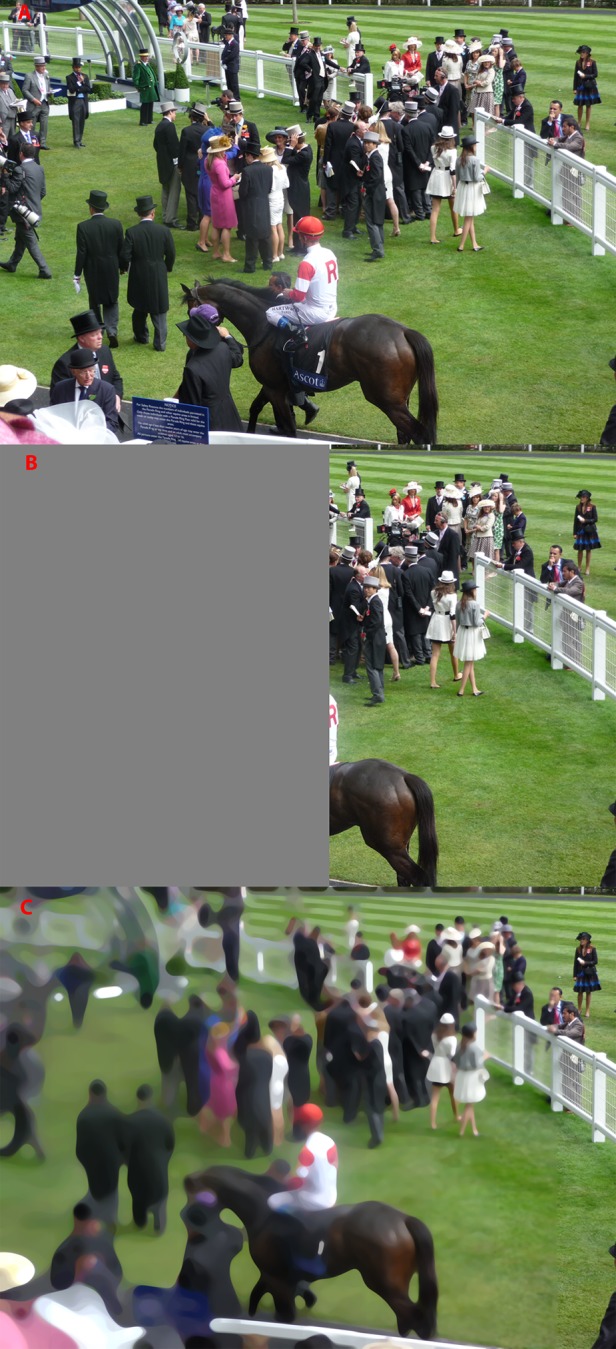
Schematic representation of how a visual scene might appear to people with left homonymous hemianopia (middle panel) and left neglect (bottom panel). Whereas hemianopia obeys the midline and affects only the contralesional visual field, neglect affects parts of the ipsilesional field in addition to the contralesional field, such that there is a lateralised bias of attention towards the side of the lesion.

Neglect should be distinguished from the related phenomenon of sensory extinction (sometimes termed visual or sensory inattention). This refers to a failure to report a contralesional stimulus only in the presence of a competing ipsilesional stimulus, when both stimuli are briefly simultaneously presented^2^ (figure 1). Neglect and extinction often coexist but can also sometimes dissociate from each other, and patients can sometimes continue to show visual extinction after their neglect has recovered. Note that neglect and extinction do not obey the vertical meridian in the way that hemianopia or quadrantanopia do, but rather represent a gradient across space. Thus, patients can still neglect or extinguish items that are *relatively* contralesional, even if they are still within the ipsilesional half of space (see figure 2 and video link below).^14^ With severe neglect, it may be very difficult to detect hemianopia; in our experience, in these circumstances careful clinical examination by confrontation, sometimes using visual threat, helps more than automated methods.

## The ‘typical’ patient

Although there are several standard tests for neglect (see below), people with moderate to severe neglect show a number of behaviours that are often clearly visible to relatives as well as clinical staff. The most severely affected patients direct their gaze towards the side of the lesion, to the point where they will not fixate on the person speaking to them. In addition, they may eat food only from one side of their plate, or pay less attention to one side when grooming, such that they shave, or apply make up to, only one side of their face (sometimes referred to as personal neglect). Neglect may also be very apparent to therapists during rehabilitation. For instance, a wheelchair user may repeatedly bump into walls and objects on the neglected side, or may omit words when reading text on the one side of the page, or misread one side of individual words (neglect dyslexia). Some patients tend not to use their contralesional limb even when there is no weakness or sensory loss: this is termed motor neglect (see table 1 for further related deficits). For descriptions from two patients and their relatives, see http://www.theguardian.com/science/video/2012/dec/23/stroke-half-world-disappear-video (also available at https://www.youtube.com/watch?v=d4FhZs-m7hA).

**Table 1:** Related impairments

Anosognosia	Unawareness of a specific deficit. Patients may be unaware of neglect and also unaware of hemiplegia.
Somatoparaphrenia	A delusional belief relating to the contralesional limbs or side of the body, such that a patient does not believe that the limb/side belongs to them.
Allochiria	A patient responds to a stimulus to one side of the body as if it had been to the other side. It can also be present in drawings where items from the contralesional side are transposed to the ipsilesional side.
Constructional apraxia	The inability to draw or copy complex diagrams. This is often associated with right hemisphere damage and persists after rightward bias has resolved. It is not related to motor apraxia.

## Tests for neglect

**Figure 3:**
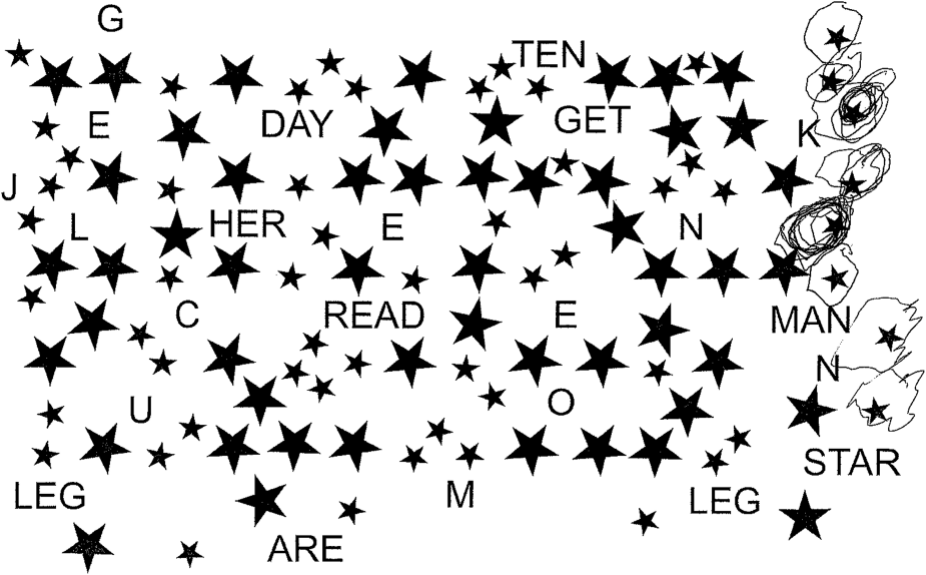
Star cancellation task from the behavioural inattention test.^27^ Patients are asked to find and mark all the small stars without marking the large stars or letters. Patients with severe neglect find targets only at the ipsilesional side of the array, even when they have unlimited time to complete the task. Patients with less severe neglect still tend to start on the right side of the array but may miss only a small number of contralesional targets.

**Figure 4:**
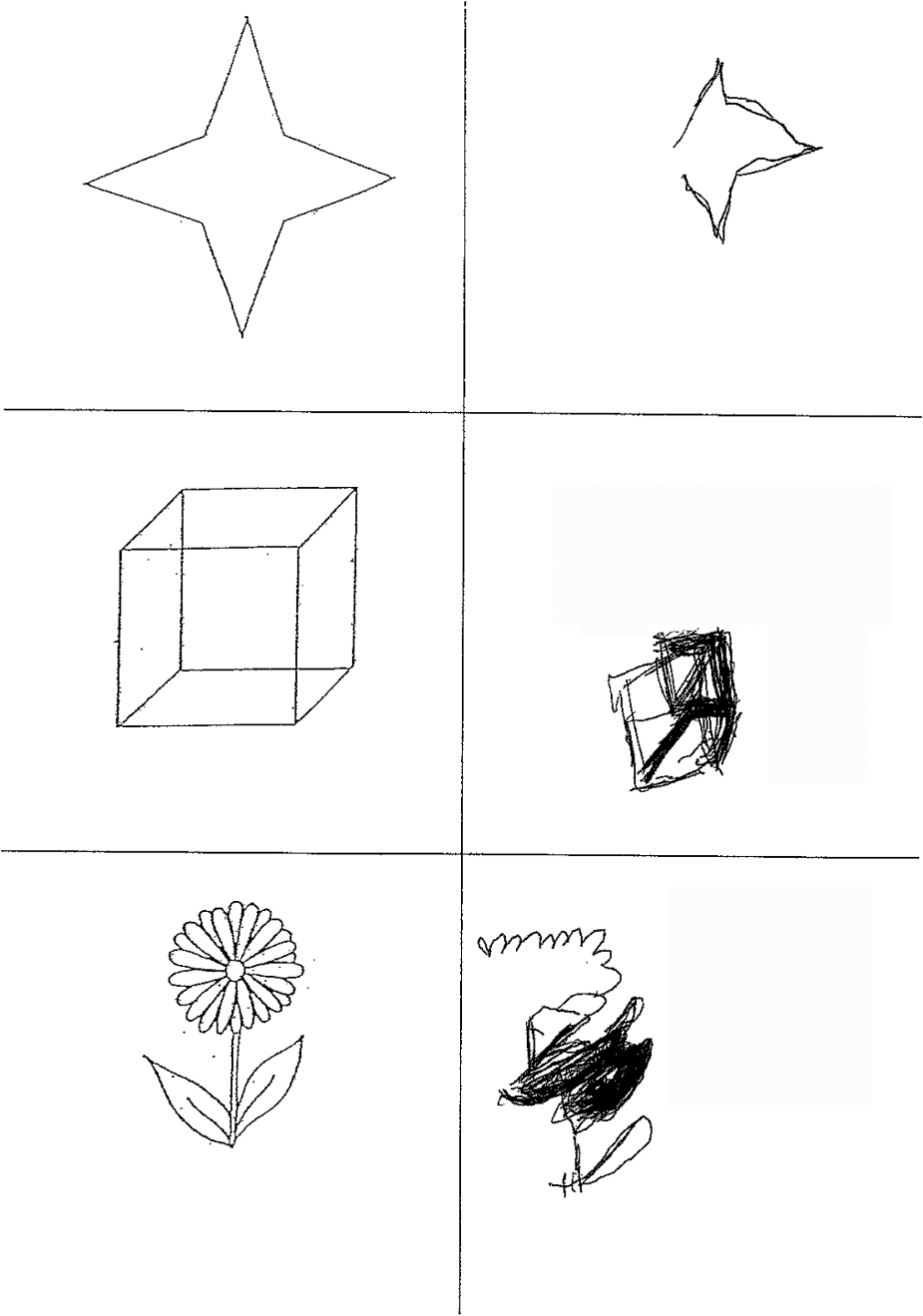
Copying task from the behavioural inattention test.^27^ The patient tends to omit the left-sided elements of each object.

Traditionally, clinicians employ pen-and-paper tasks for their ease of use. These include cancellation tests (eg, see figure 3), line bisection as well as copying (figure 4) and drawing objects. Cancellation tests are most frequently used and are singly more sensitive at detecting visuospatial neglect.^15^ These require subjects to find targets (sometimes embedded amongst distractors) on a centrally placed sheet of paper: patients with neglect tend to start at the ipsilesional edge of the page, often failing to cancel the more contralesional targets altogether. Note that denser arrays with more distractors may reveal a greater degree of neglect, although they are sometimes more difficult for a patient to do.^16^

Line bisection tasks involve marking the midpoint of one or more horizontal lines; patients with left neglect tend to err towards the right of the true centre. More complex figures may be copied or drawn from memory, with subsequent contralesional omissions or distortions of details.^17^

In addition to these pen-and-paper tasks testing for neglect in peripersonal space, there are several tests that tap into the different types of neglect behaviour. For example, personal neglect can be assessed through ecological tests where patients are asked to mime combing their hair or other grooming activities.^18^ Asking people to name items in the room around them tests for neglect in far extrapersonal space: patients with neglect often ignore objects on the left side.^19^ Patients can even be examined for representational neglect by asking them about landmarks in a familiar place (eg, the main square in their home town) and noting whether they omit items on the left side of a remembered scene or mental image.^20^ ^21^

Interestingly, an individual patient's degree of neglect can fluctuate strikingly,^22^ ^23^ even within the same day.^24^ This depends upon factors such as overall arousal, which is known to be affected by right hemisphere stroke.^13^ In addition, although many people show neglect on more than one type of task, some groups of patients show dissociations, demonstrating a lateralised bias on only one test and normal performance on others^15^ ^25^; even these characteristics may vary over time.^26^ A battery of tests is therefore more sensitive to the presence of neglect than is one single task.^27–29^ This variability probably reflects the underlying heterogeneity of each individual's cognitive deficits.^30^ Although the core deficit in neglect clearly involves a spatial bias towards ipsilesional space and away from contralesional space, most patients also have several other deficits, some of which are not spatially lateralised but may still contribute to the clinical severity of the syndrome.^7^ ^31^ This, in turn, reflects the underlying neuroanatomy, as discussed below.

A large proportion of patients recover spontaneously, in that their performance on standard tasks improves.^32^ However, this may be partially secondary to compensatory strategies during formal neuropsychological testing, which do not carry over into activities of everyday living.^33^ In fact, a growing body of evidence suggests that patients who have apparently recovered may still show impairments of attention when tested with more sophisticated tasks.^34^ ^35^

## The anatomy of neglect

Neglect most frequently follows right cerebral hemisphere damage, as a consequence of middle cerebral artery territory stroke. Although the syndrome is traditionally most closely associated with parietal lesions,^36^ most middle cerebral artery strokes affect several regions and many patients show varying combinations of parietal, temporal and frontal damage.^37^ In addition, neglect can follow subcortical stroke, although this may relate to hypoperfusion and dysfunction of overlying cortical areas.^38^ ^39^

**Figure 5:**
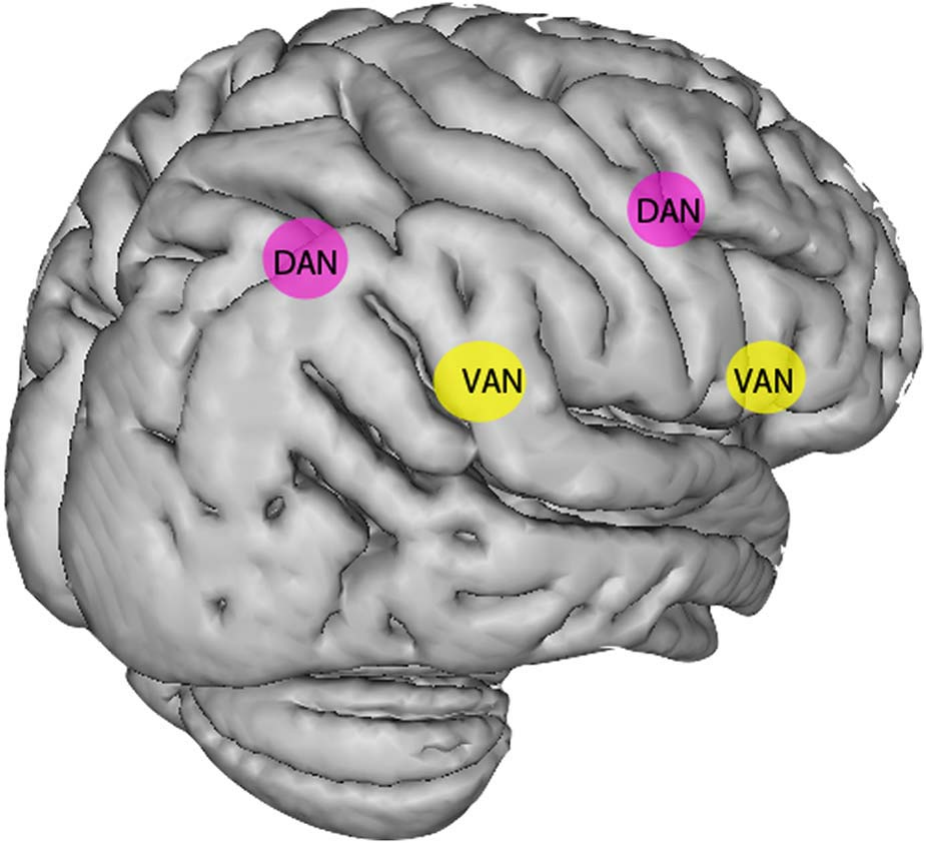
Disruption of attention networks in patients with spatial neglect. The dorsal attention network (DAN, in pink), critical to deploying spatial attention, includes the frontal eye fields anteriorly and the intraparietal sulcus posteriorly. The ventral attention network (VAN, in yellow), which is right-lateralised, includes the inferior frontal gyrus anteriorly and the temporoparietal junction posteriorly. The VAN is involved in sustained attention and arousal, as well as the reorienting of attention. Current accounts of the pathophysiology of neglect suggest that both these networks are disrupted in neglect, with the ventral network (including white matter connections) frequently being structurally damaged by a middle cerebral artery territory stroke, and the dorsal network often remaining structurally intact but showing disrupted function.^7^

There has been a great deal of controversy concerning the precise anatomy of neglect.^40^ This partly relates to the different imaging methods and time points at which patients have been tested and/or scanned.^41^ ^42^ Moreover, many of the divergent findings probably follow from the fact that neglect is a heterogeneous syndrome, and that research groups have used different tests to diagnose the presence of neglect.^43^ In fact, several studies suggest that impairments on different types of task more likely localise to different regions.^37^ ^44^ ^45^ Moreover, neglect results from damage to networks of regions involved in attention (see figure 5), and recent work has shown that it can result from damage to white matter tracts, particularly the superior longitudinal fasciculus, as well as individual cortical and subcortical regions.^46^ ^47^ At a functional level, the evidence suggests that there is an interhemispheric imbalance in patients with neglect, such that the left hemisphere is relatively overactive after a right hemisphere stroke, causing attention and eye movements to be biased rightwards.^48^ ^49^

The right frontal and parietal regions involved in spatial functions—including the deployment of attention—are also involved in several non-spatial processes such as vigilance and the ability to maintain performance on a task over an extended period of time.^50^ Thus, these non-spatial impairments often accompany, and potentially exacerbate, the key spatial features of neglect^31^ ^51^ (see box 1). The right hemisphere lateralisation of these cognitive domains also explains the increased severity and endurance of left neglect compared to right neglect. That is, right-sided strokes lead to several non-spatially lateralised cognitive deficits that exacerbate the effects of spatial bias as well as prolonging recovery.^7^ ^31^

Box 1 **Cognitive deficits in spatial neglect**

- Core deficit
  Attentional bias towards the side of the lesion
- Contributory deficits
  Arousal and vigilance
  Visuospatial working memory
  Capacity of attention resources

## Treatment of neglect

Although different approaches have been tried in neglect, including behavioural and pharmacological therapies, there is still no clear consensus as to which are most effective and which patients are most likely to respond.^52^ ^53^ One particular difficulty in treating this population is that many patients show anosognosia for neglect (see table 1) and hence are unaware of their deficit. This unawareness negatively impacts upon their final outcome.^54^

The main approach, repeatedly pursued in the treatment and rehabilitation of neglect, involves directly addressing the core deficit of neglect and attempting to reorient attention towards the neglected side. Visual scanning therapy is widely used in rehabilitating patients with neglect. It mainly involves encouraging them to explore the left side of space, often with the help of visual cues.^55^ However, there is little persuasive evidence that it has significant long-term effects.^33^

Vestibular stimulation via caloric irrigation or galvanic stimulation may successfully reorient attention and transiently reduce personal, extrapersonal and even representational neglect.^56^ ^57^ Tactile vibration of the left side of the neck has similar effects and may be most effective when combined with other techniques.^58^ In addition, limb activation through passive and active movements of the neglected limb may sometimes help.^59^

One technique that appears to give longer-lasting changes in neglect involves optical prisms.^60^ Patients with left neglect initially wear prisms that displace the visual scene *rightward*, causing them to misreach to the right of a target. After repeated pointing, and once they have adapted such that they reach accurately, the prisms are removed, leaving patients with a leftward pointing error. Such prism adaptation therapy considerably benefits multiple aspects of neglect.^61^ Although prism adaptation seems to be among the most promising treatments of the last two decades, the evidence from randomised controlled trials is equivocal. It may be that appropriate patient selection is key to effective intervention.^62–65^

A more recent treatment involves brain stimulation techniques. Several studies have suggested that the core deficit in neglect relates to asymmetrical activation of each cerebral hemisphere, such that the lesioned hemisphere is underactive compared with the intact side, with a subsequent imbalanced deployment of spatial attention.^66^ Thus, this asymmetry could be successfully treated either by activating the lesioned hemisphere or inhibiting the overactive contralesioned hemisphere. Early studies with transcranial magnetic stimulation, delivered as theta burst stimulation to inhibit the left hemisphere, suggest that it can accelerate recovery of left neglect^67^ and reduce disability with respect to activities of daily living.^68^

A very different approach to the treatment of neglect aims to improve the contributing cognitive deficits that are not directly related to spatial bias. This can be achieved through non-pharmacological means, such as using alerting tones to boost arousal.^69^ It has also been attempted with a noradrenergic agent, guanfacine, which improved visual search in neglect patients without frontal damage.^70^ Several investigators have also studied dopaminergic therapies since dopaminergic pathway lesions can induce neglect-like behaviour in animal experiments.^71^ ^72^ Although these studies gave conflicting results,^73^ ^74^ the most recent trial involving 16 patients showed that rotigotine, a dopamine agonist, improved cancellation task performance as well as boosting selective attention.^75^

A less obvious line of research has aimed to improve neglect by enhancing motivation.^76^ Anecdotally, task performance has been shown to improve with the incorporation of a monetary goal^77^ and standard contemporary therapy also involves clearly-defined goals. However, there are now empirical studies showing improvement of neglect using motivational and rewarding stimuli in different forms, including anticipated monetary gain,^78^ altering task instruction,^79^ passively listening to pleasant music^80^ and playing a musical sequence.^81^ Although these probably exert their effects via multiple mechanisms, reward processing and music listening have been repeatedly linked to dopaminergic systems,^82^ ^83^ and dopamine may play a crucial role in motivational interventions.

## Conclusions

A key theme running through this review is the syndromic nature of neglect and its underlying heterogeneity. Given this, no one therapeutic approach will be appropriate for all patients, and a key aim for future work will be the careful delineation of cognitive deficits and their individual responsiveness to specific treatments. One further important consideration relates to our understanding of recovery from neglect. It is critical to determine whether residual deficits following apparent recovery on standard tests relate to ongoing symptoms, and how much they impact on activities of daily living.
